# Optogenetic quantification of cardiac excitability and electrical coupling in intact hearts to explain cardiac arrhythmia initiation

**DOI:** 10.1126/sciadv.adt4103

**Published:** 2025-02-28

**Authors:** Judith S. Langen, Patrick M. Boyle, Daniela Malan, Philipp Sasse

**Affiliations:** ^1^ Institute of Physiology I, Medical Faculty, University of Bonn, Bonn, Germany.; ^2^ Department of Bioengineering, University of Washington, Seattle, Washington, USA.; ^3^ Institute for Stem Cell and Regenerative Medicine, University of Washington, Seattle, Washington, USA.; ^4^ Center for Cardiovascular Biology, University of Washington, Seattle, Washington, USA.; ^5^ eScience Institute, University of Washington, Seattle, Washington, USA.

## Abstract

Increased cardiac excitability and reduced electrical coupling promote cardiac arrhythmia and can be quantified by input resistance (*R*_m_), pacing threshold (*I*_thr_), and cardiac space constant (λ). However, their measurement in the heart was not feasible because the required homogenous current injection cannot be performed with electrical stimulation. We overcame this problem by optogenetic current injection into all illuminated cardiomyocytes of mouse hearts in different action potential phases. Precisely triggered and patterned illumination enabled measuring *R*_m_ and λ, which both were smallest at diastole. Pharmacological and depolarization-induced reduction of inwardly rectifying K^+^ currents (*I*_K1_), gap junction block, and cardiac infarction reduced *I*_thr_, showing the importance of high *I*_K1_ density and intact cardiomyocyte coupling for preventing arrhythmia initiation. Combining optogenetic current injection and computer simulations was used to classify pro- and anti-arrhythmic mechanisms based on their effects on *R*_m_ and *I*_thr_ and allowed to quantify *I*_K1_ inward rectification in the intact heart, identifying reduced *I*_K1_ rectification as anti-arrhythmic concept.

## Teaser

Combining optogenetic current injection with simulations allows for quantification of arrhythmia mechanisms in intact hearts.

## INTRODUCTION

Due to electrical coupling of cardiomyocytes through gap junctions, the heart is a functional electrical syncytium, which is important not only for rapid conduction of electrical excitation but also for prevention of pathological activity. In cardiac tissue, excitatory impulses act as sources of depolarizing current for adjacent repolarized tissue (sink), and the source current density must be sufficient to depolarize the sink to its activation threshold or propagation will fail (*1*). Consequently, in well-coupled healthy myocardium, a pathological afterdepolarization in an individual cardiomyocyte (source) results in electrotonic current flow to the neighboring repolarized cardiomyocytes (sink), failing to induce a potentially dangerous premature ventricular contraction (PVC) (*2*). Thus, loss of coupling plays a critical role in arrhythmogenesis, not only by slowing conduction but also by reducing the protective sink and, accordingly, decreasing the source current density required to trigger a PVC (*1*). In addition, repolarizing outward currents influence cardiac electrical stability by amplifying sink effects and counteracting potential afterdepolarizations. At rest, the major contributor to these outward currents is the inwardly rectifying K^+^ current (*I*_K1_), which stabilizes resting membrane potential (RMP) and shapes final repolarization (*3*). Its outward K^+^ current is regulated by voltage-dependent block of the pore by intracellular positively charged polyamines, resulting in a reduced conductance at depolarized membrane potentials (*3*), which is important for fast action potential (AP) upstroke and conduction velocity.

Ventricular arrhythmia due to afterdepolarization-induced PVCs is the leading cause of sudden cardiac death (*4*). Quantification of cardiac excitability and cell-to-cell coupling in the intact heart is crucial for investigating mechanisms of arrhythmia initiation and maintenance. Three separate but linked parameters are important to consider: (i) the input resistance (*R*_m_) of a cardiomyocyte, which describes the membrane potential change in response to a subthreshold current injection. Low diastolic *R*_m_ is attributed to the high conductance of *I*_K1_. Thus, if a pathological depolarizing current arises, then the resulting (after)depolarization, given mathematically by Ohm’s law as the product of *R*_m_ and current amplitude, will be small. However, if *I*_K1_ is pathologically reduced, as in heart failure (*5*, *6*), then *R*_m_ increases and the voltage change caused by the same current will be larger, potentially triggering a PVC (*7*). (ii) The pacing threshold as a measure of the current density sufficient to trigger an AP in a single cell (*I*_thr_), which correlates inversely with the PVC vulnerability. (iii) The space constant (λ), which describes the spread of subthreshold depolarization in space via electrotonic current flow to neighboring cardiomyocytes, quantifying electrical coupling.

Because many pathological conditions, including heart failure and myocardial infarction, involve increased occurrence of cardiac arrhythmias (*5*, *8*), we propose that quantification of susceptibility to afterdepolarizations and PVCs by *R*_m_ and *I*_thr_ as well as electrical coupling in intact hearts by λ is important to improve our understanding of arrhythmia mechanisms.

Previous studies often focused on disease-specific changes in currents by patch clamp and gene expression analysis. However, the changes in any individual current can be relatively small and their interaction complex, making the consequences for arrhythmia initiation difficult to predict. For example, *I*_K1_ reduction, which can be seen in heart failure patients (*5*, *6*), has been reported to have both pro- and anti-arrhythmic potential depending on the pathophysiologic context (*9*, *10*), highlighting the need to consider the integrated effects of all ionic currents. Furthermore, several drugs are multichannel blockers and their integrated pro- or anti-arrhythmic consequences on cardiac excitability are difficult to predict. Moreover, in contrast to patch clamp experiments, measurement of *R*_m_, *I*_thr_, and λ can be performed in intact hearts, which is important because many pathological conditions such as myocardial infarction or changes in electrical coupling cannot be sufficiently reproduced in single cells. Thus, we propose that quantification of functional parameters such as *R*_m_, *I*_thr_, and λ in the intact heart is required to provide a mechanistic framework for detailed understanding of the electrophysiological changes that promote arrhythmias and to derive therapeutic concepts.

Until recently, it was not possible to precisely measure *R*_m_, *I*_thr_, and λ in the intact heart because homogenous depolarization of cardiomyocytes is required, which was not feasible by single-cell current injection in the syncytium of electrically coupled cardiomyocytes or by extracellular electrical stimulation. Thus, previous approaches were limited to isolated cells and small-tissue preparations or used nonhomogeneous extracellular field stimulation, which induces not only depolarization of cardiac tissue but also areas of hyperpolarization at virtual anodes (*11*). To overcome these limitations, we have developed optogenetic current clamp to directly quantify *R*_m_, *I*_thr_, and λ in the intact heart during the cardiac cycle with high temporal resolution. This is enabled by sharp microelectrode recording of membrane potential combined with precisely timed, confined illumination of hearts expressing the light-gated cation channel channelrhodopsin-2 (ChR2), which leads to the required current injection in all illuminated cardiomyocytes (*12*).

## RESULTS

### Optogenetic determination of *R*_m_ in intact hearts

**Fig. 1.**
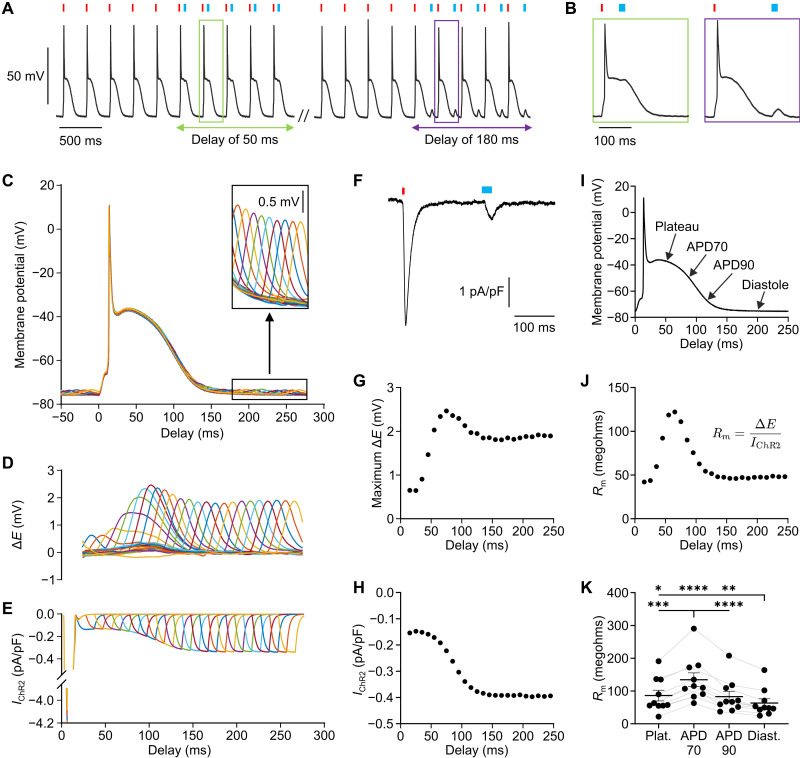
Optogenetic determination of input resistance (*R*_m_) in intact hearts. (**A** and **B**) Representative AP trace with pacing (red, ~350 μW/mm^2^, 5 to 10 ms) and subthreshold (blue, 15 to 40 μW/mm^2^, 20 ms) light stimulation (465 nm). (**C**) Averaged APs with second subthreshold light pulses of delays between 10 and 240 ms. (**D** and **E**) Membrane potential change (D, Δ*E*) calculated as difference between averaged APs with and without subthreshold light pulse and corresponding ChR2 current (E, *I*_ChR2_) computed by a ChR2 gating model for the different delays. (**F**) ChR2 current of a ventricular cardiomyocyte evoked by high (red, 315.5 μW/mm^2^) and subthreshold (blue, 13.6 μW/mm^2^) illumination analog to the stimulation in (A) to (E). (**G** to **J**) Representative averaged AP (I) and corresponding values of *R*_m_ (J) calculated as ratio of maximum membrane potential change (G) and ChR2 current (H) [(A) to (J), from one representative heart or cardiomyocyte]. (**K**) *R*_m_ at AP plateau, 70 and 90% of repolarization (APD70 and APD90) and diastole [repeated measures one-way analysis of variance (ANOVA), Tukey’s multiple comparisons posttest, *N* = 10, *P* < 0.0001]. Means ± SEM. *P < 0.05, **P < 0.01, ***P < 0.001, and ****P < 0.0001.

We developed an optogenetic current clamp method to measure *R*_m_ in the intact heart and in different AP phases. Precisely timed current injection in all cardiomyocytes of the left ventricular free wall was performed by epicardial illumination of Langendorff-perfused mouse hearts expressing the light-gated ion channel ChR2 (*12*). High-intensity light pulses were used to pace hearts with a fixed cycle length of 275 ms followed by low-intensity subthreshold light pulses with defined delays (Fig. 1, A and B). Membrane potential was measured by sharp microelectrode recording and averaged over multiple cycles to reduce noise (Fig. 1C). Subthreshold depolarization (Δ*E*) was calculated as maximum difference between the averaged APs with and without subthreshold illumination (Fig. 1, D and G). The injected ChR2 current (*I*_ChR2_) was computed by a ChR2 gating model (Fig. 1, E and H) (*13*), which was calibrated to match measurements from patch clamp experiments of ventricular cardiomyocytes expressing ChR2 (Fig. 1F, *n* = 7). *R*_m_ was calculated according to Ohm’s law as *R*_m_ = Δ*E*/*I*_ChR2_ for each delay (Fig. 1, I and J) and was significantly smaller during diastole (Fig. 1K; 63.3 ± 13.1 megohms = 100%) compared to the plateau (137.4 ± 10.3%), first (APD70, 227.9 ± 11.2%), and second (APD90, 133.4 ± 3.9%) phases of final repolarization. Furthermore, *R*_m_ at APD70 was significantly higher than *R*_m_ during other AP phases, suggesting that the heart is particularly vulnerable to afterdepolarizations triggering PVCs in this first phase of final repolarization (Fig. 1K).

### Quantification of *I*_K1_ contribution to *R*_m_ and *I*_thr_

**Fig. 2.**
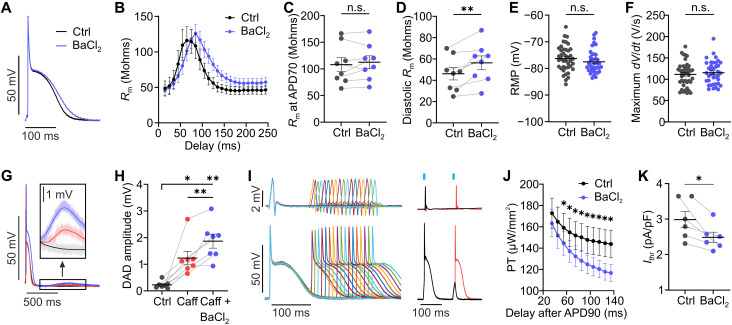
Effect of *I*_K1_ reduction on cardiac excitability. (**A** and **B**) Representative, averaged APs (A) and *R*_m_ for different delays (B) of the subthreshold light pulse with and without BaCl_2_ (10 μM). (**C** to **F**) Quantification of the effect of *I*_K1_ block on *R*_m_ during the first phase of final repolarization [(C), at APD70, two-tailed, paired *t* test, *N* = 8, *P* = 0.39] and diastole [(D), two-tailed, paired *t* test, *N* = 8, *P* = 0.0016], RMP [(E), two-tailed, unpaired *t* test, *N* = 4, *n* = 38-41, *p* = 0.25], and maximum upstroke velocity [(F), maximum *dV*/*dt*, two-tailed, unpaired *t* test, *N* = 4, *n* = 38 to 41, *P* = 0.53]. (**G**) DADs resulting from fast pacing (cycle length of 70 to 140 ms) without (black), with caffeine (1 mM, red), and with BaCl_2_ additionally to caffeine (blue). (**H**) Statistical analysis of DAD amplitude (repeated measures one-way ANOVA, Tukey’s multiple comparisons posttest, *N* = 7, *P* = 0.0003). (**I**) Representative AP traces of a S1-S2 protocol (left, last S1 showed) followed by a premature stimulus (S2) of variable delay (120 to 260 ms) and traces of the transition from sub (black) to supra-threshold (red) optical stimulation (right). (**J** and **K**) Thresholds for optogenetic pacing (PT) for different delays of S2 (10-ms binning) after APD90 (J) and statistics of diastolic *I*_thr_ determined 135 ms after APD90 [(K), two-tailed, paired *t* test, *N* = 6, *P* = 0.02] with and without BaCl_2_. Means ± SEM. *P < 0.05 and **P < 0.01; n.s., not significant; Mohms, megohms.

To determine the contribution of *I*_K1_ to *R*_m_ in different AP phases, the *I*_K1_ blocker BaCl_2_ was applied at low concentration, resulting in a small AP prolongation (Fig. 2A) and an increase in diastolic *R*_m_ of 23.6 ± 4.6% (Fig. 2, B and D) but without affecting *R*_m_ during the first phase of final repolarization (APD70, Fig. 2C). This concentration of BaCl_2_ did not depolarize cells (Fig. 2E) or change the maximum AP upstroke velocity (Fig. 2F). To show that *I*_K1_ reduction can underlie the vulnerability of the heart to afterdepolarizations, we analyzed the amplitude of spontaneous diastolic delayed afterdepolarizations (DADs). DADs were promoted by fast pacing to induce Ca^2+^ overload in the intracellular stores, which alone did not produce DADs (Fig. 2, G and H, black). However, in combination with caffeine, which sensitizes ryanodine receptors to release Ca^2+^, spontaneous DADs of an amplitude of 1.23 ± 0.25 mV (Fig. 2, G and H, red) could be observed. Administration of BaCl_2_ increased DAD amplitude to 1.87 ± 0.27 mV (Fig. 2, G and H, blue). Calculation of the currents underlying these DADs using Ohm’s law (*I*_DAD_ = *E*_DAD_/*R*_m_) showed almost identical values (caffeine, 32.9 pA; caffeine + BaCl_2_, 33.2 pA). Thus, the change in DAD amplitude induced by BaCl_2_ could be completely explained by the increase in *R*_m_. To investigate whether the increased *R*_m_ also corresponds with an increased susceptibility to PVCs, we determined the threshold for optogenetic pacing (in microwatts per square millimeter) in different AP phases (Fig. 2I) and calculated diastolic *I*_thr_ (in picoamperes per picofarad), which was significantly reduced by 15.77 ± 3.42% in the presence of BaCl_2_ (Fig. 2K).

**Fig. 3.**
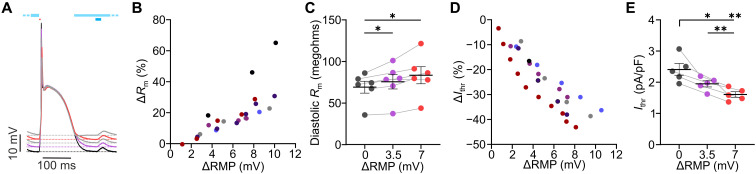
Effect of RMP depolarization on cardiac excitability. (**A**) Representative, averaged, paced (red bar) AP traces without (black) and with low-intensity illumination during the diastole (9.4 to 33.6 μW/mm^2^, light blue bar) and subthreshold pulses to determine *R*_m_ (dark blue bar). (**B** to **E**) Relationship between depolarization of RMP (ΔRMP) and change of *R*_m_ (B, Δ*R*_m_) or *I*_thr_ (D, Δ*I*_thr_) with different colors indicating individual hearts. Statistical analysis of diastolic *R*_m_ [(C), *N* = 6, *P* = 0.021] and *I*_thr_ [(E), *N* = 5, *P* = 0.0015] for 3.5- and 7-mV RMP depolarization (repeated measures one-way ANOVA, Tukey’s multiple comparisons posttest). Means ± SEM. *P < 0.05 and **P < 0.01.

Beyond *I*_K1_ reduction, depolarization of the RMP has been discussed as another critical factor promoting cardiac arrhythmia in ischemic heart disease and heart failure (*5*, *6*). To investigate the effect on *R*_m_ and *I*_thr_, sustained, stepwise increasing, low-intensity illumination was applied during diastole (Fig. 3A, light blue bar), which increased RMP up to ~10 mV. In addition, pacing and subthreshold light pulses were applied to determine *I*_thr_ and *R*_m_, respectively (Fig. 3A, red and dark blue bars). RMP depolarization significantly increased diastolic *R*_m_ (Fig. 3, B and C) and decreased *I*_thr_ (Fig. 3, D and E).

### Modeling of ionic currents underlying cardiac excitability

**Fig. 4.**
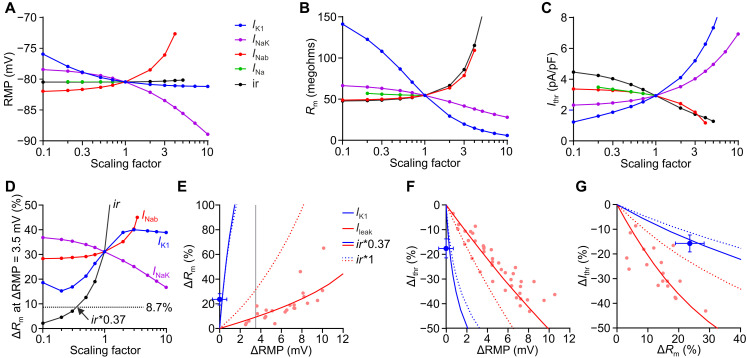
Contribution of diastolic ion currents to excitability in a computational model of a mouse ventricular cardiomyocyte. (**A** to **C**) Effect of scaling *I*_K1_, Na^+^,K^+^-ATPase current (*I*_NaK_), sodium background current (*I*_Nab_), voltage-dependent sodium current (*I*_Na_), and inward rectification (*ir*) of *I*_K1_ on RMP (A), diastolic *R*_m_ (B), and *I*_thr_ (C). (**D**) Relative change in *R*_m_ in response to RMP depolarization of 3.5 mV by adding a cation background current (*I*_leak_) mimicking *I*_ChR2_ for different scaling factors of diastolic currents. Dotted, black line indicates the experimentally measured increase in *R*_m_ of 8.7%. (**E** to **G**) Relationships between relative changes in RMP, *R*_m_, and *I*_thr_ predicted by the model with ir*1 (dotted lines) and ir*0.37 (solid lines) for reduction of *I*_K1_ (blue) and increase in *I*_leak_ (red). Gray line indicates effect of 3.5 mV RMP depolarization on *R*_m_ (E). Experimental data from Figs. 2 (blue, means ± SEM) and 3 (red) are shown as dots.

To investigate the relative contributions of individual currents to cardiac excitability and RMP in the diastole, we conducted computer simulations allowing for modification of ion current densities and *I*_K1_ inward rectification. The simulations were performed using a model of a mouse ventricular cardiomyocyte (*14*) incorporating the ChR2 gating model (*13*). To quantify RMP, *R*_m_, and *I*_thr_ in the model, an analysis framework was developed in openCARP to mimic experimental conditions. We found that decreasing *I*_K1_ or Na+- and K+-dependent adenosine triphosphatase (Na^+^,K^+^-ATPase) current (*I*_NaK_) as well as increasing Na^+^ background current (*I*_Nab_) led to depolarization of the RMP (Fig. 4A), an increase in *R*_m_ (Fig. 4B), and a decrease in *I*_thr_ (Fig. 4C), and vice versa. An increase in *I*_K1_ inward rectification led to similar effects on *R*_m_ and *I*_thr_ without affecting the RMP (Fig. 4, A to C).

To compare experimental data with simulation predictions, we modeled the effect of RMP depolarization by adding a cation background current (*I*_leak_) mimicking *I*_ChR2_. Experimentally, a RMP depolarization of 3.5 mV led to an increase in *R*_m_ of 8.7% (Fig. 3, B and C), and, in simulations, the same RMP depolarization resulted in an *R*_m_ increase of ~30% (Fig. 4E, intersection of dashed red and gray line). To reduce this discrepancy, we tested scaling of the currents involved during diastole (Fig. 4D). Only a reduction of the *I*_K1_ inward rectification parameter *ir* (Eq. 2) to 37%, but not up- or downscaling of current densities, was able to explain the experimental depolarization-induced change in *R*_m_ (Fig. 4D, dotted, black line). Moreover, the calibrated (Fig. 4, E to G, ir*0.37, red solid lines), but not the original model (Fig. 4, E to G, ir*1, red dotted lines), perfectly predicted the relationships between changes of RMP, *R*_m_, and *I*_thr_ of the experimental data (dots) from optogenetic RMP depolarization. The experimental data of *I*_K1_ reduction by BaCl_2_ (Fig. 4, E to G, blue dots) matched both the original (blue dotted lines) and the calibrated (blue solid lines) model and, thus, could not be used to identify *I*_K1_ inward rectification. Furthermore, the interdependency of RMP, *R*_m_, and *I*_thr_ can be used to discriminate between increased excitability due to moderate *I*_K1_ block, which reduced *I*_thr_ and increased *R*_m_ without affecting RMP (Fig. 4, E and F, blue) and increased excitability due to increased leak current, which additionally depolarizes RMP (Fig. 4, E and F, red) and has a stronger effect on *I*_thr_ than on *R*_m_ (Fig. 4G).

**Fig. 5.**
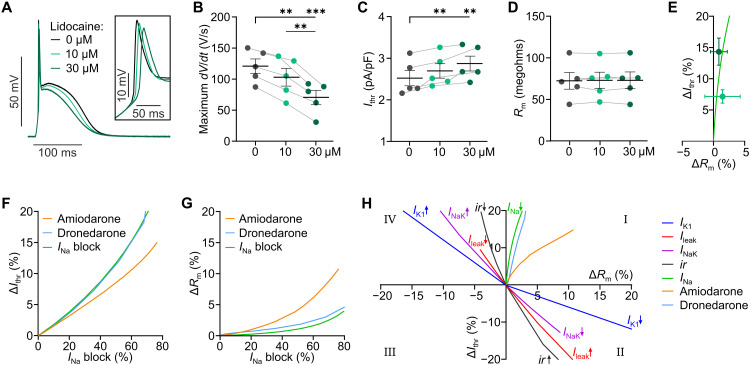
Effect of Na^+^ and multichannel blockers on cardiac excitability. (**A** to **D**) Representative, averaged APs (A), maximum upstroke velocity [(B), maximum *dV/dt*, *N* = 5, *P* < 0.0001], *I*_thr_ [(C), *N* = 5, *P* = 0.0022], and diastolic *R*_m_ [(D), *N* = 5, *P* = 0.73] without and with the sodium channel blocker lidocaine (10, 30 μM, repeated measures one-way ANOVA, Tukey’s multiple comparisons posttest). (**E**) Relationship between change of *R*_m_ (Δ*R*_m_) and *I*_thr_ (Δ*I*_thr_) resulting from sodium current reduction in simulations (solid line) and experiments (dots). (**F** and **G**) Simulated relationship between degree of *I*_Na_ block and increase in pacing threshold (F) and *R*_m_ (G) for increasing concentrations of the multichannel blockers amiodarone and dronedarone and isolated *I*_Na_ block. (**H**) Summary of the relationship between Δ*R*_m_ and Δ*I*_thr_ for isolated modifications of indicated ion channels, I_K1_ inward rectification (*ir*), and the multichannel blockers from simulations in the calibrated cardiomyocyte model. Means ± SEM. **P < 0.01 and ***P < 0.001.

Patients with pro-arrhythmic increase in cardiac excitability can benefit from sodium channel blockers like lidocaine. As expected, lidocaine led to a reduction of the AP upstroke velocity and amplitude (Fig. 5, A and B) and significantly reduced *I*_thr_ (Fig. 5C) without affecting *R*_m_ (Fig. 5D). This was confirmed in simulations, which showed a similar increase in *I*_thr_ (Fig. 5, E and F, green) and only a small increase in *R*_m_ (Fig. 5, E and G, green). In addition, we simulated effects of the anti-arrhythmic drugs amiodarone and dronedarone, which not only block sodium currents but also affect repolarizing K^+^ currents, Ca^2+^ currents, and the Na^+^,K^+^-ATPase, using the degree of block reported in Loewe *et al*. (*15*). These simulations showed that dronedarone behaved similar to lidocaine with minor effects on *R*_m_, whereas amiodarone increased *R*_m_ (Fig. 5G). This suggests a relevant block of stabilizing K^+^ conductances, which explains the smaller effect on *I*_thr_ compared to the other two blockers (Fig. 5F).

Our experiments suggest that the interdependency of *R*_m_ and *I*_thr_ is highly specific for different ion channel blockers (Figs. 4G and 5E). To classify effects of drugs or pathological conditions affecting cardiac excitability, we simulated their effects on the relationship between change in *R*_m_ and *I*_thr_ (Fig. 5H, quadrants I to IV). Quadrant I describes the effect of multichannel blockers, which reduce excitability by blocking Na^+^ channels, thus increasing *I*_thr_; this effect may be counteracted by blocking K^+^ currents, leading to increased *R*_m_ (e.g., amiodarone). Quadrant II shows pathological situations with reduced *I*_K1_ or Na^+^,K^+^-ATPase as well as increased leak currents or *I*_K1_ inward rectification, leading to an increase in *R*_m_ and a decrease in *I*_thr_. These effects could be reversed by the anti-arrhythmic conditions shown in quadrant IV, which will protect from PVCs not only by increasing *I*_thr_ but also by decreasing *R*_m_.

### Optogenetic determination of λ in the intact heart

**Fig. 6.**
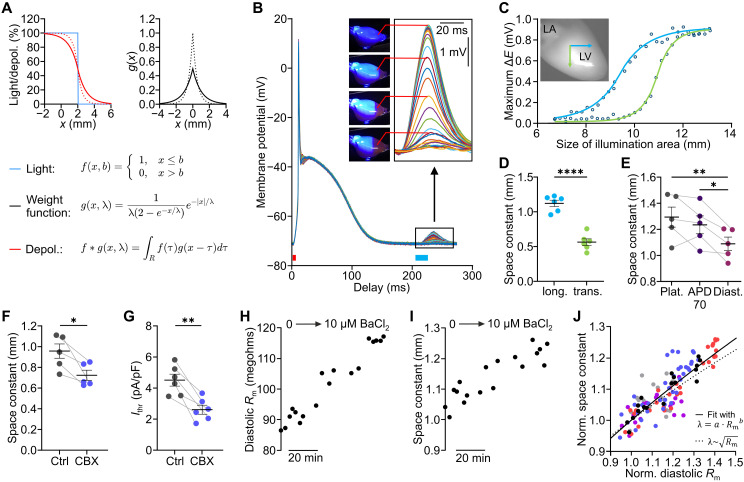
Optogenetic determination of cardiac space constant (λ) in the intact heart. (**A**) Concept for determination of λ. Theoretical example of depolarization in one-dimensional space (left, red) resulting from illumination restricted in space (blue) with high (solid, 1 mm) and low (dotted, 0.5 mm) λ. The magnitude of depolarization is calculated by convolution (*f* ∗ *g*) of the distribution of light (*f*) with a weight function (*g*, right) which depends on λ (solid, 1 mm; dotted, 0.5 mm). (**B**) Averaged APs with different illumination sizes of the subthreshold light pulses (blue bar, 20 ms) applied after a global pacing pulse (red bar, 5 to 10 ms). (**C** and **D**) Relationship between subthreshold membrane potential change (Δ*E*) and size of illumination area fitted with the convolution function *f* ∗ *g* [(C); LA, left atrium; LV, left ventricle] and diastolic λ obtained from fitting λ as parameter of *g* upon decreasing the illumination area in longitudinal (blue) and transverse (green) direction of fiber orientation [(D), two-tailed, unpaired *t* test, *N* = 6, *P* < 0.0001]. (**E**) Longitudinal λ at plateau (Plat.), 70% of repolarization (APD70), and diastole (Diast.; repeated measures one-way ANOVA, Tukey’s multiple comparisons posttest, *N* = 5, *P* = 0.008). (**F** and **G**) Diastolic, longitudinal λ [(F), *N* = 5, *P* = 0.032] and *I*_thr_ [(G), *N* = 6, *P* = 0.0028] without and with the gap junction blocker carbenoxolone (CBX; 10 μM, two-tailed, paired *t* test). (**H** to **J**) Change in diastolic *R*_m_ (H) and λ (I) during successive increase of BaCl_2_ concentration up to 10 μM in one representative heart and normalized changes in *R*_m_ and λ (J) fitted with a power function (solid black, *a* = 1.002, 99% confidence interval: [0.986, 1.018], *b* = 0.572, 99% confidence interval: [0.487, 0.656]). Square root relationship shown with dotted line. Colors indicate different hearts. Means ± SEM. *P < 0.05, **P < 0.01, and ****P < 0.0001.

To quantify electrical coupling between cardiomyocytes in the intact heart, we developed an optogenetic method to measure λ by localized subthreshold stimulation. First, a mathematical description of the magnitude of depolarization depending on the distribution of light and the electrical coupling was derived. Without electrical coupling, the regional distribution of depolarization in response to optogenetic stimulation equals the distribution of light (Fig. 6A, blue function *f*). However, electrical coupling between the cells leads to a sigmoidal distribution of depolarization along the boundary of the illumination area (Fig. 6A, red function *f* ∗ *g*), which can be explained by electrotonic current flow between the adjacent cells. The resulting magnitude of depolarization in space can be described by convolution of the light function *f* with a weight function *g*, which describes the exponential spread of a subthreshold stimulus in one dimension and depends on λ (Fig. 6A).

Experimentally, λ was determined by successively decreasing the illumination area, which led to an increased distance to the recording site (Fig. 6B, inset, recording site in red) and, therefore, to a reduction of the subthreshold depolarization (Fig. 6, B and C). Because λ depends on the fiber orientation (*16*), we measured λ in longitudinal and transverse fiber directions (Fig. 6C, blue and green arrows, respectively). Fitting the relationship between size of illumination area and magnitude of subthreshold depolarization with the convolution function *f* ∗ *g* (Fig. 6C) could be used to reliably determine λ, which was significantly larger in longitudinal (Fig. 6D, blue, 1.12 ± 0.05 mm) than in transverse direction of fiber orientation (Fig. 6D, green, 0.56 ± 0.05 mm). In addition, we measured the longitudinal λ at different time points during the cardiac cycle and found that the diastolic λ (Fig. 6E, 1.09 ± 0.05 mm) was significantly smaller compared to λ at the first phase of final repolarization (Fig. 6E, APD70, 1.23 ± 0.07 mm) or plateau (Fig. 6E, 1.29 ± 0.08 mm). To validate this method, we applied the gap junction blocker carbenoxolone that significantly reduced diastolic λ by 22.3 ± 5.7% (Fig. 6F). Carbenoxolone also decreased *I*_thr_ by 41.3 ± 6.2% (Fig. 6G), suggesting a critical role of uncoupling in arrhythmia initiation.

Cable theory predicts a square root relationship between λ and *R*_m_ ($\lambda ∼\sqrt{R_{m}}$) (*17*), but this has not been experimentally proven in intact hearts. Thus, we applied increasing concentrations of the *I*_K1_ blocker BaCl_2_ and measured the increase in diastolic *R*_m_ and λ at identical time points (Fig. 6, H and I). Their relationship was quantified by fitting with a power function (Fig. 6J, black solid line) and was consistent with the square root relation (black dotted line).

### Effect of electrical uncoupling on cardiac excitability

**Fig. 7.**
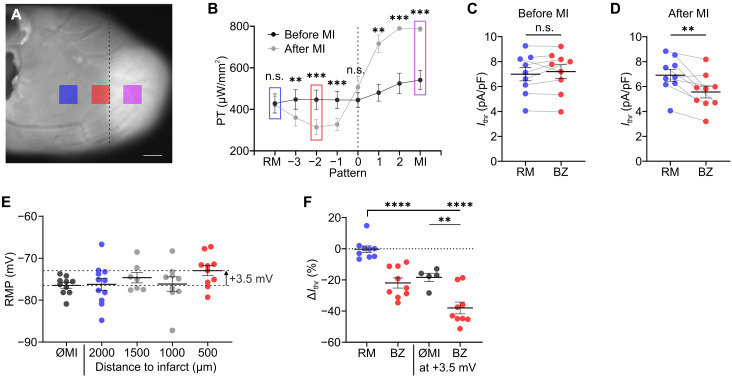
Increased cardiac excitability after acute myocardial infarction. (**A** and **B**) Image of the left ventricle (A) after cryoinfarction (white) at the heart apex (scale bar, 1 mm) and pacing threshold (PT) before and after infarction for different regions (B). The border of infarction is indicated with dashed line, and pattern of remote myocardium (RM), border zone (BZ; pattern −2), and infarction (MI) are highlighted in blue, red, and purple, respectively [(A) and (B)]. (**C** and **D**) Statistical analysis of *I*_thr_ before [(C), two-tailed, paired *t* test, *N* = 9, *P* = 0.38] and after infarction [(D), two-tailed, paired *t* test, *N* = 9, *P* = 0.0037]. (**E**) RMP before (∅MI) and after cryoinfarction recorded at different distances from the border of infarction (ordinary one-way ANOVA, *N* = 2 to 3, *n* = 9 to 11, *P* = 0.27). (**F**) Change in *I*_thr_ (Δ*I*_thr_) of RM and BZ without and with assuming 3.5-mV RMP depolarization after infarction compared to Δ*I*_thr_ due to 3.5-mV RMP depolarization alone (∅MI, data from Fig. 3E) (ordinary one-way ANOVA, *N* = 5 to 9, *P* < 0.0001, calculated on the basis of values in picoamperes per picofarad). Means ± SEM. **P < 0.01, ***P < 0.001, and ****P < 0.0001.

Myocardial infarctions increase the risk of PVCs triggering potential lethal ventricular arrhythmia. To quantify the mechanisms involved, we determined *I*_thr_ after acute cardiac infarction in the remote, healthy myocardium (Fig. 7A, blue), the infarcted area with loss of cardiomyocytes (Fig. 7A, purple), and the border zone between both regions (Fig. 7A, red) by successively moving the illumination pattern from the remote area to the infarction (Fig. 7A). After infarction, *I*_thr_ was significantly decreased near the border of infarction (Fig. 7B, patterns −3 to −1) and increased in the infarcted area (Fig. 7B, patterns 1, 2, and MI). Furthermore, after infarction, *I*_thr_ was significantly reduced in the border zone compared to the remote myocardium (Fig. 7D), whereas there was no significant difference between the two locations before infarction (Fig. 7C). A decreased *I*_thr_ could be due to electrical uncoupling between infarcted and adjacent myocardium (Fig. 6G) but also due to RMP depolarization (Fig. 3), both leading to a reduced electrical sink. To discriminate between these two effects, we measured RMP at different distances from the infarcted area. Near the border of the infarct, RMP was depolarized up to 3.5 mV (although not significant) compared to RMP before infarction (Fig. 7E), which would by itself decrease *I*_thr_ by 18.4 ± 2.6% (Figs. 3E and 7F). After infarction, *I*_thr_ was unchanged in the remote myocardium (0 ± 2.1%) but significantly reduced in the border zone (−22 ± 3.2%, Fig. 7F). Assuming 3.5-mV RMP depolarization, the calculated *I*_thr_ was further reduced in the border zone (−37.9 ± 3.8%), which was significantly larger than the effect of 3.5-mV depolarization alone (−18.4 ± 3.6, Fig. 7F). Thus, the reduction in *I*_thr_ in the border zone of ~38% can be only partly explained by a depolarized RMP (~18%), and the other ~20% must be due to other reasons, e.g., because of electrical uncoupling and loss of cardiomyocytes in the infarcted area (see discussion).

## DISCUSSION

Reduced electrical coupling and increased excitability are important factors in myocardial infarction, heart failure, arrhythmia, and sudden cardiac death. We suggest that quantification of functional parameters such as *R*_m_, *I*_thr_, and λ in the intact heart is essential for detailed understanding of electrophysiological changes that promote arrhythmias.

Our light-induced current clamp method enabled to determine these functional arrhythmia parameters in the intact heart. The diastolic *R*_m_ in mouse hearts (~60 megohms, Fig. 1K) was in the range of those measured by current injection in isolated rat (40 to 60 megohms) (*18*, *19*) and guinea pig (~10 megohms) (*20*) ventricular cardiomyocytes. *R*_m_ in canine papillary muscle (20 to 225 kilohms) (*21*) or tissue islands of this muscle (0.25 to 1.5 megohms) (*21*) was much smaller, presumably because part of the injected current was lost due to electrotonic current flow to neighboring cardiomyocytes, leading to *R*_m_ underestimation. In contrast, optogenetic stimulation allows for light-induced current injection in all cardiomyocytes expressing ChR2. Because we used epicardial blue light illumination, which can be absorbed and backscattered by cardiac tissue, the transmural light attenuation could lead to an overestimation of *I*_ChR2_ at greater depths, resulting in an underestimation of *R*_m_. However, we expect that this effect is minor because most of the measurements were performed within the first 200 μm from the epicardium. Furthermore, for statistical comparison of interventions on *R*_m_ and *I*_thr_, we only compared effects of cells with <80-μm difference in depths, thus reducing a potential error in the relative changes reported.

Despite differences in plateau and repolarization phase between mice and humans, we believe that our approach of quantifying diastolic electrical stability in mice has translational value for human arrhythmia, because *I*_K1_ as major contributor to stabilization of RMP is very similar between mice and human (*22*–*24*). In contrast to mice with physiological cycle length of 100 ms in vivo (600 beats per minute), humans have a stable diastolic potential for at least 500 ms at rest. To mimic this, we used a suitable pacing rate (275 ms), resulting in stable diastolic intervals of >100 ms (Fig. 1).

The use of patterned, optogenetic current clamp and mathematical convolution allowed to determine λ in longitudinal (~1.1 mm) and transverse (~0.6 mm) direction of fiber orientation (Fig. 6D), which is in range of those determined using multiple electrodes (*25*–*27*) or optical mapping, although the latter required reduction of excitability by high extracellular K^+^ concentrations (*17*, *28*). The epicardial space constant measured in our study might be slightly underestimated in the longitudinal and overestimated in the transverse direction because of ChR2-induced subthreshold depolarizations in deeper layers with transmural rotation of fiber orientations. However, this effect is also present when using epicardial voltage mapping to determine conduction velocity or length constant. In the context of the present study, we deployed cell-scale computational models to facilitate careful analysis of how specific ionic currents affect cardiac excitation. Conceivably, a more elaborate computational model [e.g., a three-dimensional wedge of ventricular tissue with realistic fiber orientations, as in (*29*)] could be used to resolve the abovementioned subtle inaccuracy of our λ measurements. However, this would greatly increase the analytical and computational complexity of the computational work and jeopardize our ability to use the models for their intended purpose in this study. Nevertheless, our work provides a framework for potential future applications in this direction.

Mild reduction of gap junctional coupling decreased λ by 22% and reduced *I*_thr_ by more than the double (46%). These results underline the importance of electrical coupling in the prevention of PVCs (*30*) and are in line with increased arrhythmogenesis in cardiac fibrosis, ischemia-induced gap junctional uncoupling, or cardiomyocyte loss after myocardial infarction, which all reduce the electrical sink of the surrounding myocardium and, thereby, facilitate PVCs (*1*, *31*). Similarly, in our experiments, *I*_thr_ was decreased after cardiac infarction by ~40% in the border zone (Fig. 7F), of which about half could be attributed to a RMP depolarization of ~3.5 mV. This could be due to elevated extracellular K^+^ released from dying cardiomyocytes in the infarcted area (*32*) or due to increased diastolic leak currents (see discussion below). The other half (Fig. 7F) could be due to loss of electrical sink by gap junctional uncoupling, loss of viable myocardium, or reduction in diastolic K^+^ currents (*33*). However, because the cryoinjury infarct model generates precise, well-localized infarctions without ischemic or hypoxic conditions in the surrounding tissue, which could activate ATP-sensitive K^+^ currents or reduce diastolic K^+^ currents such as *I*_K1_ (*33*, *34*), we do not expect changes in outward K^+^ currents contributing to the decreased *I*_thr_ in the border zone. In the future, measurements of *R*_m_, *I*_thr_, and λ under hypoxic condition will be important to investigate the effect of hypoxia and ischemia on cardiac excitability and coupling.

Beyond diastolic values, we were able to determine *R*_m_ and λ during the cardiac cycle using triggered optogenetic current clamp. *R*_m_ was lowest in the diastole reflecting the importance of *I*_K1_ for preventing diastolic PVCs and highest during first phase of final repolarization (APD70), indicating a vulnerability to PVCs in this phase. Quantitatively, *R*_m_ was ~2-fold larger at APD70 compared to diastole (Fig. 1K), which is less than the difference described in single cardiomyocytes (~200-fold) (*20*, *35*). This discrepancy may be due to nonsynchronous repolarization in intact hearts, which can reduce *R*_m_ during repolarization because some cardiomyocytes are still in plateau or already at diastole, dynamically acting as electrical sink in a well-coupled syncytium, an effect further promoted by the increased λ in the first phase of final repolarization (Fig. 6E). Furthermore, increased electrical coupling during repolarization reduces gradients of repolarization and, accordingly, lowers the chance that a PVC induces reentry during this highly vulnerable phase in which *R*_m_ is high.

Heart failure and myocardial infarction are associated with increased PVC susceptibility (*5*–*7*, *9*) and ~4- to 9-mV depolarization of RMP (*36*, *37*), which has been attributed to reduced *I*_K1_. However, in our experiments, partial block of *I*_K1_ by 10 μM BaCl_2_, which was shown to reduce *I*_K1_ similarly as in heart failure (*9*), did not depolarize RMP (Fig. 2E), presumably because RMP was close to the K^+^ reversal potential. Thus, the RMP depolarization seen in heart failure may be rather due to pathological depolarizing, diastolic leak currents, which could result from a leaky mode of the Na^+^,K^+^-ATPase at reduced adenosine 5′-triphosphate (ATP) levels (*38*–*41*), from a sustained, tetrodotoxin-sensitive Na^+^ current recorded during hypoxia (*40*, *42*, *43*), or from connexin hemichannels activated by ischemia (*44*). To quantify excitability in response to RMP depolarization, we experimentally added a leak current by diastolic, low-intensity illumination. Leak-induced depolarization increased *R*_m_, potentially due to voltage-dependent polyamine block of *I*_K1_ (*3*). Quantitatively, the effect of 7-mV depolarization on *R*_m_ (Fig. 3C) was comparable to the effect of pharmacological *I*_K1_ reduction (Fig. 2D) but with much higher *I*_thr_ reduction (Figs. 2K and 3E). This suggests that RMP depolarization increases PVC vulnerability not only by increasing *R*_m_ but also by reducing the distance from RMP to threshold for AP initiation.

Comparing experimentally measured relationships between RMP, *R*_m_, and *I*_thr_ with mathematical simulations showed that the increase in *R*_m_ due to a depolarizing leak current was largely overestimated by the original model (Fig. 4E). This discrepancy could only be resolved by reducing *I*_K1_ inward rectification, but not by up- or downscaling of current densities (Fig. 4D). Thus, combining RMP depolarization with optogenetic current clamp allows to quantify the voltage-dependent inward rectification of *I*_K1_ in the intact heart, which before was only possible by patch clamp of single cells. *I*_K1_ rectification is an essential parameter because it quantifies how much the polyamine block of *I*_K1_ induced by RMP depolarization reduces the sink counteracting PVC mechanisms.

Kir2.1 is the main isoform of I_K1_ in mouse ventricle (*45*), and its inward rectification was much stronger in isolated mouse cardiomyocytes (*ir* of ~0.04 to 0.09, Eq. 2, Materials and Methods) (*14*, *46*, *47*) and heterologous expression systems (*ir* of ~0.09) (*48*) than in our experiments in the intact mouse heart (*ir* of 0.0332, Fig. 4D). This discrepancy could be due to higher ATP levels in Langendorff-perfused hearts (8 mM) (*49*) compared to patch clamp solutions (2 to 3 mM) (*14*, *47*), which binds polyamines and, thereby, reduces *I*_K1_ rectification (*50*). Our simulations show the importance of low *I*_K1_ rectification to reduce cardiac excitability (Figs. 4C and 5H), and we speculate that low ATP levels in cardiac disease may abolish this protective mechanism by increasing free polyamines levels and consequently *I*_K1_ rectification. Thus, additionally to increasing *I*_K1_ density, suggested before to treat arrhythmia (*51*–*53*), we propose reduction of its inward rectification, e.g., by direct interaction with the channel or by reducing ornithine decarboxylase activity to lower polyamine levels, as therapeutic concept preventing PVCs. It was shown that the anti-arrhythmic drug flecainide not only blocks Na^+^ channels but also reduces Kir2.1 rectification (*54*), which would protect from functional *I*_K1_ loss due to depolarization or ATP depletion. Only optogenetic current clamp can measure the integrated effects of such multichannel drugs on cardiac excitability in the intact heart.

In summary, PVC vulnerability depends on the surrounding sink mainly generated by repolarizing outward K^+^ currents counteracting potential afterdepolarizations. The magnitude of electrical sink of a single cell can be quantified by *R*_m_, and the spatial extent of sink due to coupling between cardiomyocytes can be quantified by λ. Furthermore, the probability of afterdepolarizations triggering an AP depends on the membrane potential distance to the activation threshold of voltage-dependent Na^+^ channels as well as their availability and current density, which can be measured by *I*_thr_. Experimentally, the latter was confirmed by application of the *I*_Na_ blocker lidocaine that significantly increased *I*_thr_ without affecting *R*_m_ (Fig. 5, C and D), underlining *I*_Na_ as major determinant of excitability and source without affecting the sink. Clinically used *I*_Na_ or multichannel blockers reduce PVCs by increasing *I*_thr_ (Fig. 5H, quadrant I) without counteracting pathologically increased *R*_m_ that is often underlying the increased PVC vulnerability in cardiac diseases (Fig. 5H, quadrant II). Thus, we suggest that better therapeutic concepts could be *I*_K1_ enhancement or reduction of its inward rectification (Fig. 5H, quadrant IV), which both not only increase *I*_thr_ but also decrease *R*_m_. We conclude that characterizing pathological pro-arrhythmic conditions and anti-arrhythmic therapies by analyzing their effects on *R*_m_, λ, and *I*_thr_ provides a more comprehensive assessment compared to approaches focusing only on effects on ion channels. Our optogenetic current clamp method is feasible in both computational simulations (*55*–*57*) and experimental preparations, although the latter is now limited to transgenic mouse models. However, the development of larger animal models by AAV gene transfer (*58*), combined with this methodology, is important to improve our understanding of cardiac arrhythmias and to test therapeutic strategies such as reduction of *I*_K1_ rectification.

## MATERIALS AND METHODS

### Animal model

All experiments were in accordance with the European Guideline for animal experiments 2010/63/EU. Experiments for determination of RMP and afterdepolarization amplitude were performed with 11 male and 7 female CD-1 wild-type hearts. For all other experiments, 33 male and 43 female transgenic hearts from a previously established mouse line (*12*, *55*) expressing ChR2 (H134R mutation) and enhanced yellow fluorescent protein under control of the chicken–β-actin promotor and backcrossed at least 10 generations on a CD-1 genetic background were used.

### Microelectrode recording

Mice were euthanized by cervical dislocation. Hearts were explanted and retrogradely perfused in Langendorff configuration at a constant pressure of 110 cmH_2_O with Tyrode’s solution [142 mM NaCl, 5.4 mM KCl, 1.8 mM CaCl_2_, 2 mM MgCl_2_, 10 mM glucose, and 10 mM Hepes (pH 7.4), adjusted with NaOH, 100% O_2_] containing 10 μM blebbistatin (Enzo Life Science, TargetMol) to inhibit contractions. The perfusion solution was heated to 42°C, resulting in a temperature of the mouse myocardium of 35° ± 1°C, and the flow was measured with a flow meter (SLF3S-1300F, Sensirion). Chemicals and reagents were purchased from Sigma-Aldrich, if not specified otherwise. To measure membrane potential, sharp microelectrodes (15 to 40 megohms, 1B100F-4, World Precision Instruments, pulled with P-1000 Micropipette Puller, Sutter Instrument) filled with 3 M KCl were inserted into the left ventricular free wall and pushed forward through the myocardial wall using a fast piezoelectric actuator with position output (Sensapex uMp, ~5- to 10-μm steps). The actual depth was determined considering the position output and penetration angle (45°) of the microelectrode. Membrane potential was recorded with a microelectrode amplifier (BA03S, NPI electronics) and a PowerLab recording system (PowerLab 8/30, LabChart software, ADInstruments, 10-kHz sampling rate). Cells with an AP amplitude smaller than 70 mV were excluded from analysis.

### Optogenetic stimulation

For optogenetic stimulation, patterned illumination of the left ventricular free wall was performed with a 465-nm light-emitting diode (LED; LEDMOD HP, Omicron Laserage) and a digital mirror device (Polygon400-G, Mightex) coupled to a macroscope (THT, Scimedia) with a 1× objective [MVPLAPO, Olympus, 0.25 numerical aperture (NA)]. All optical stimuli were applied as square pulses. Light intensities were calibrated using a power meter (PM100A, S130C, Thorlabs). Both atria were removed to reduce spontaneous heart rate and allow for optogenetic pacing with a cycle length of 275 ms. The threshold for optogenetic pacing was measured by restricting the illumination to a circular area of 11.2 mm^2^ (Figs. 2-5) or 6.2 mm^2^ (Fig. 6) or a square area of ~1 mm^2^ (Fig. 7) and applying an S1-S2 protocol consisting of six S1 light pulses at a cycle length of 275 ms (10 ms, ~350 μW/mm^2^) followed by a S2 stimulus of variable delay and light intensity. The pacing threshold was defined by the lowest light intensity of the S2 pulse that allowed for successful pacing in five consecutive S1-S2 iterations. If not otherwise indicated, then pacing thresholds were determined during diastole with a S2 interval of 265 ms. Optogenetic RMP depolarization (Fig. 3) was achieved by using low-intensity light of a second 460-nm LED (LED Hub, Omicron Laserage) and a light guide collimated with a lens for uniform illumination of the left ventricular free wall. In some experiments, a bipolar surface electrogram was recorded with a silver chloride electrode placed at the base of the left ventricle and a metal spoon under the apex using a bio-amplifier recording system (ML 136, PowerLab 8/30).

### RMP determination

Wild-type hearts were electrically paced (1 to 10 mA, 0.5 ms, biphasic, cycle length of 275 ms) via a monopolar platinum/iridium microelectrode (Science Products, PI2PT30.1B10) placed on the epicardium. If the sharp microelectrode touched the epicardial surface, then the offset of the microelectrode amplifier was set to zero and the electrode resistance was measured. The electrode resistance was determined at each cell, and recording was discarded if the electrode resistance changed >10 megohms from the initial value, indicating a break or clotting of the tip. RMP was determined by using the peak analysis module of LabChart and averaging over 10 s.

### Patch clamp

Adult ventricular cardiomyocytes were enzymatically isolated from ChR2 transgenic mice as described previously (*12*, *59*) and plated at low density on laminin-coated (0.1%) coverslips in external solution [142 mM NaCl, 5.4 mM KCl, 1.8 mM CaCl_2_, 2 mM MgCl_2_, 10 mM glucose, and 10 mM Hepes (pH 7.4), adjusted with NaOH, 22°C]. Patch-clamp recordings were made with an EPC10 amplifier and the Patchmaster software (Heka) using the whole-cell configuration. Patch pipettes (3 to 5 megohms) were filled with internal solution containing 50 mM KCl, 80 mM K-asparatate, 1 mM MgCl_2_, 3 mM MgATP, 10 mM EGTA, 10 mM Hepes (pH 7.2), adjusted with KOH. Light-induced ChR2 currents were measured in voltage clamp mode at −75.4 mV by illuminating with a light guide–coupled 465-nm LED (LED Hub, Omicron Laserage) with attenuation filters through a 20× objective (S-Fluor, 0.75 NA) on Eclipse TI-2E microscope (Nikon) using high-intensity illumination (315 μW/mm^2^, 5 ms) followed by a low-intensity subthreshold light pulse (13.6 μW/mm^2^, 20 ms) with a delay of 200 ms.

### Protocol for induction of afterdepolarizations

To induce spontaneous afterdepolarizations, we used a stimulation protocol consisting of fast electrical pacing (cycle length of 70 to 140 ms for 40 cycles), followed by a break of 10 s where afterdepolarizations were analyzed. After the break, hearts were paced with a cycle length of 215 ms for at least 30 cycles before repeating the protocol. If the spontaneous heart rate was too fast to allow for analysis of afterdepolarizations, then 10 μM carbachol was applied (in one of the seven hearts). Moving average filtering over 100 ms was performed on all membrane potential traces prior to analysis of afterdepolarization amplitude.

### Model of cryoinfarction

To investigate cardiac excitability after acute myocardial infarction, an acute cryoinjury infarct model was used that generates precise infarction size and allows for a well-defined localization of the infarcted area. The cryoinfarction was produced by applying a liquid nitrogen cooled copper probe three times for 15 s to the apical part of the free left ventricular wall (*60*). Pacing thresholds of 10 illumination pattern (~1 mm by 1 mm), which were successively moved from base to apex (330- to 660-μm steps), were measured before and after cryoinfarction. If the maximum light intensity (~800 μW/mm^2^) did not lead to successful pacing, then *I*_thr_ was set to the maximum technical available light intensity.

### Simulation of ChR2 currents

ChR2 currents were calculated using a previously described ChR2 gating model (*13*), which was implemented in MATLAB (2020b, MathWorks) and calibrated to match the peak current of the subthreshold light pulse measured in patch clamp experiments of adult cardiomyocytes expressing ChR2 (−0.45 ± 0.03 pA/pF at 13.6 μW/mm^2^, *n* = 7). The ChR2 current for calculation of *R*_m_ was converted in pA by assuming a membrane capacity of 100 pF per cardiomyocyte. To precisely calculate the ChR2 current during the cardiac cycle to determine *I*_thr_ and *R*_m_, the applied, calibrated light intensities and the averaged membrane potential trace of each experiment were used because the ChR2 photocurrents depend on the membrane potential (Fig. 1, E and H). To compensate for offset changes during an experiment, membrane potential traces were shifted so that the diastolic potential matched the RMP measured under control conditions (−75.4 ± 0.6 mV, *n* = 30, *N* = 3). If the RMP was optogenetically depolarized, then the diastolic potential was additionally corrected by the constant light-induced RMP depolarization (Fig. 3). To account for peak current desensitization, the simulations started with 15 s of pre-pacing (membrane potential trace of AP without subthreshold illumination, cycle length of 275 ms) before the AP cycle of interest was simulated.

### Computational simulation of cardiac electrophysiology

Single-cell simulations were performed using a murine ventricular cardiomyocyte model (*14*). Computational experiments were conducted using the openCARP simulation environment (version 8.2) (*61*). To allow for optogenetic determination of *R*_m_ and pacing threshold, the calibrated ChR2 gating model described above (*13*) was integrated into the cardiomyocyte model. Analogous to the experiments, *R*_m_ was determined in the simulations via delivery of a subthreshold light pulse (20 ms, 15 μW/mm^2^) after simulating at least 10 AP cycles (cycle length of 275 ms) induced by optogenetic pacing. The pacing threshold was defined as lowest light intensity at which 10 of 10 light pulses (10 ms) induced APs. RMP was defined as the minimum membrane potential after simulating 10 AP cycles. The RMP was depolarized by adding a cation background current (*I*_leak_) to the cardiomyocyte model, mimicking the ChR2 current in the experiments with a K^+^ conductance of 0.5 relative to Na^+^ conductance (*62*). *I*_leak_ was calculated as

$$I_{\text{leak}}=g_{\text{leak}}\left(V-E_{\text{Na}}\right)-0.5\;\left(V-E_{K}\right)$$ (1)

where *V* is the membrane potential, *g*_leak_ is a scaling factor, and *E*_Na_ and *E*_K_ are the Na^+^ and K^+^ reversal potentials, respectively. Simulations for Fig. 4 (A to D) were performed without modification of the *I*_K1_ inward rectification parameter *ir*. The experimentally measured increase of *R*_m_ (8.7% at 3.5-mV RMP depolarization) could be achieved by reducing *ir* to 37% (from initial 0.0896 to 0.0332) in the *I*_K1_ equation of the model, which was subsequently used (Figs. 4, E to G, and 5, E to H)

$$I_{K1}=0.2938\left(\frac{{\left[K^{+}\right]}_{o}}{{\left[K^{+}\right]}_{o}+210.0}\right)\left(\frac{V-E_{K}}{1+e^{\text{ir}\;\left(V-E_{K}\right)}}\right)$$ (2)

Values of *R*_m_, RMP, and *I*_thr_ were only used if the model was stable (i.e., the simulated cell returned to RMP after each AP). Calculation of percentage changes of pacing thresholds was based on ChR2 photocurrents in picoamperes per picofarad. Simulation of the effects of the multichannel blockers amiodarone and dronedarone was adapted from previously described half maximal inhibitory concentrations and Hill coefficients (*15*).

The openCARP code for determining *R*_m_, *I*_thr_, and inward rectification including a modified model with *I*_leak_ is available as Supplementary Materials.

### Data analysis and statistics

The analysis of the data, including filtering, calculation of ChR2 current and *R*_m_, detection of different AP phases and parameters, determination of afterdepolarization amplitude, and fitting of λ by the method of least squares, was performed with MATLAB R2020b with the optimization toolbox (MathWorks). For analysis of cycle-dependent *R*_m_, the diastole was defined between 200 and 240 ms after AP initiation, the first and second phases of final repolarization as time points of 70% (APD70) and 90% (APD90) of repolarization, respectively, and plateau timing as 0.5*APD70. To determine *R*_m_ and pacing threshold at 3.5- and 7-mV RMP depolarization, data were linearly interpolated (Fig. 3, C and E). λ was obtained by fitting the maximum measured subthreshold membrane potential change of different light pattern with

$$f*g\left(x,\lambda \right)=\int_{\mathbb{R}}f\left(\tau \right)g\left(x-\tau \right)d\tau$$ (3)

where

$$f\left(x,b\right)=\left\{ \begin{array}{c} 1,\;x\leq b\\ 0,\;x>b \end{array}\right.$$ (4)

is the function of light depending on the position of the microelectrode b and

$$g\left(x,\lambda \right)=\frac{1}{\lambda \left(2-e^{-x∕\lambda }\right)}e^{-|x|∕\lambda }$$ (5)

the weight function. Fits of λ with a mean square error larger than 0.3 mm^2^ were excluded from analysis. Cycle-dependent λ was measured 200 ms after AP initiation (diastole), at APD70 (repolarization), and 50 ms after AP initiation (plateau).

Statistics were calculated with GraphPad Prism 8 (GraphPad Software). *P* values less than 0.05 were considered statistically significant and are indicated by *P < 0.05, **P < 0.01, ***P < 0.001, and ****P < 0.0001 in the figures. Each dot represents an independent experiment with *n* and *N* values indicating the number of cells and hearts, respectively. Data are shown as means ± SEM. Hearts were excluded from analysis if their spontaneous heart rate was faster than the required cycle length or if the flow resulting from the perfusion at constant pressure of 110 cmH_2_O was larger than 4 ml/min, indicating a lesion of the aorta. For statistical comparison of *R*_m_, only cells with less than 80-μm difference in microelectrode penetration depth were considered for paired analysis to exclude confounding effects of light attenuation. The least squares fit for the relationship between *R*_m_ and λ was obtained using the lmfit package in Python, yielding a maximum-likelihood estimate as well as parameter confidence intervals using an *F* test (*63*).
