# Supplementary material for: Optogenetic quantification of cardiac excitability and electrical coupling in intact hearts to explain cardiac arrhythmia initiation
Source: Sci Adv. 2025 Feb 28;11(9):eadt4103. doi: 10.1126/sciadv.adt4103 (PMC11870084; doi:10.1126/sciadv.adt4103)
Supplement: Supplementary file 1 — Legend for data S1 [file sciadv.adt4103_sm.pdf]

Science Advances
AAAS

Supplementary Materials for

Optogenetic quantification of cardiac excitability and electrical coupling in intact hearts to explain cardiac arrhythmia initiation

Judith S. Langen *et al.*

Corresponding author: Philipp Sasse, philipp.sasse@uni-bonn.de

Sci. Adv. **11**, eadt4103 (2025)
DOI: 10.1126/sciadv.adt4103

The PDF file includes:

Legend for data S1

Other Supplementary Material for this manuscript includes the following:

Data S1

Data S1. (separate file)

The openCARP code including instructions for determining R_m, I_{thr}, and ir and a modified model with I_{leak}.
